# Comparison of the effect of albumin with platelet-rich fibrin (Alb-PRF) gel and hyaluronic acid gel injection on interdental papilla reconstruction: A randomized clinical trial

**DOI:** 10.34172/japid.2024.018

**Published:** 2024-09-11

**Authors:** Bardia Vadiati Saberi, Ali Khalighi Sigaroudi, Mobina Kamani, Elahe Rafiei

**Affiliations:** ^1^Dental Sciences Research Center, Department of Periodontics, School of Dentistry, Guilan University of Medical Sciences, Rasht, Iran; ^2^Dental Sciences Research Center, Department of Oral and Maxillofacial Surgery, School of Dentistry, Guilan University of Medical Sciences, Rasht, Iran; ^3^Department of Periodontics, School of Dentistry, Arak University of Medical Sciences, Arak, Iran; ^4^Research and Technology, Guilan University of Medical Sciences, Rasht, Iran

**Keywords:** Black triangle, Hyaluronic acid, Interdental papilla reconstruction, Non-surgical treatment, Platelet-rich fibrin

## Abstract

**Background.:**

The interdental papilla plays a crucial role in the esthetic of the smile. Papilla reconstruction surgery is one of the most unpredictable periodontal procedures. This study compared the effect of the non-surgical application of a commercial hyaluronic acid (HA) gel with an autogenous gel named "albumin with platelet-rich fibrin" (Alb-PRF) on interdental papilla reconstruction.

**Methods.:**

This trial was conducted on 46 incomplete interdental papillae with class I or II defects. The papillae were randomly divided into two experimental groups, and commercial HA or Alb-PRF (prepared from the patient’s peripheral blood) was injected into the papillae twice at a 21-day interval. Three and six months after the intervention, photographs were taken from the black triangles and the black surfaces compared to each other. Patient satisfaction and dental plaque index were also evaluated at the end of the study.

**Results.:**

The black triangles’ surfaces significantly decreased over time in both experimental groups (*P*<0.001). However, there were no significant differences between the two groups (*P*=0.994). The intervention resulted in the patients’ relative satisfaction with the treatment results in both groups. However, the difference between the two groups was not statistically significant (*P*=0.965). In addition, no statistically significant differences were observed in plaque index between the two groups (*P*=0.566).

**Conclusion.:**

HA or Alb-PRF injection into the incomplete interdental papilla may restore the soft tissue to a great extent and lead to patient satisfaction by reducing the area of black triangles that threaten esthetics; however, more studies are needed.

## Introduction

 The interdental papilla around natural teeth is crucial in protecting periodontal structures and health. The presence or absence of the papilla is a serious concern for dentists and patients.^1^ A complete papilla in the anterior region of the maxilla is the key to esthetics.^2^ The interdental black triangle is considered the third esthetic problem in dentistry, after caries and crown margins.^1^ An open gingival embrasure will cause complications such as food retention, increased accumulation of microbial plaque, reduced self-cleansing, and periodontal disease. Also, the papilla will help phonetics. The papilla defect can probably cause a lack of self-confidence during communication.^2^

 Reconstruction of the interdental papilla has limited potential compared to other parts of the gingiva.^3^ Papilla loss is multifactorial, and treatment requires a comprehensive approach to correct causative factors. Due to the specific blood supply of the papilla^2^ and its distinct cellular and molecular properties compared to other parts of the gingiva,^3^ reconstruction of the papilla is one of the most difficult regenerative periodontal treatments. In the past, the common treatment of missing papillae was only mucogingival surgery.^4^ Among the surgical techniques, autogenous subepithelial connective tissue graft, the gold standard treatment, provided stable, predictable, and beautiful results with wounds and discomfort at the donor site.^5^

 In 2010, Becker et al^6^ used hyaluronic acid (HA) injection to treat gingival recession for the first time and reported promising results. In fact, HA was able to solve the problem of papilla degeneration and black triangle generation by stimulating fibroblastic migration and fibrogenesis.^7,8^

 In several studies, the injection of HA into the interdental papilla has been investigated, and the favorable results have been maintained for at least six months.^9-11^ Today, most HA fillers are synthetic. The cross-linking process is used to increase the time of absorption of these materials, which might be responsible for causing allergic reactions after using such products.^12^ Considering the high sensitivity of the papilla and reports of complications such as swelling, discoloration, and burning sensation due to the use of filler materials^13^ and their high cost, it seems necessary to find an inexpensive autogenous alternative that reconstructs the papillary soft tissue without invasiveness.

 Platelet-rich fibrin (PRF) has been proposed as an autologous biological material with a rich growth factor content. However, it is not always applicable due to limitations such as rapid absorption (two weeks). So far, few studies have used injectable platelet-rich fibrin (i-PRF) for gingival augmentation, with acceptable results.^14,15^ According to recent studies, heating the platelet-poor plasma (PPP) (top layer in the centrifuged blood tube) produces albumin gel, increasing the absorption time of this biological substance from two weeks to more than four months. In this method introduced by Fujioka-Kobayashi et al,^16^ peripheral blood is collected in a plastic tube and centrifuged with a defined protocol. Then, albumin gel is prepared and mixed with liquid PRF, and albumin with platelet-rich fibrin (Alb-PRF) gel is prepared. This biocompatible biologic product with a long degradation period may be able to accelerate papilla regeneration by the continuous release of growth factors.^16^ It can be used as a simple and inexpensive treatment for incomplete interdental papillae.

 This study evaluated and compared the effects of “HA” and “Alb-PRF” gel injections on the interdental black triangles’ surfaces, patient satisfaction, and microbial plaque index in the treated areas in six months.

## Methods

 The present randomized clinical trial with a parallel design was conducted on 46 incomplete interdental papillae around natural teeth in the esthetic zone of 10 volunteers referred to the Specialty Department of Periodontics, Guilan University of Medical Sciences in 1400. This clinical trial was approved by the Ethics Committee of Guilan University of Medical Sciences (IR.GUMS.REC.1400.604) and the Iranian Registry of Clinical Trials (IRCT20130813014350N4).

 The volunteers aged 20‒75 years had at least one incomplete papilla with class I or II defects of the Nordland and Tarnow^17^ classification, a probing depth of ≤ 4 mm, contact point-to-bone crest distance of ≤ 7 mm, and gingival and plaque index of 0‒1. The participants signed a written informed consent form to be included in the study. Patients with gingivitis or periodontitis, diabetes, connective tissue diseases, or other systemic diseases affecting periodontal tissues and pregnant or lactating women were excluded. Also, people with a history of periodontal surgery in the last six months, a history of allergic reactions, smoking, or taking drugs that induce gingival overgrowth were excluded.

 The volunteers were excluded from the study in case of inflammatory or allergic complications, severe pain, or infection due to the intervention. Also, if the participants became pregnant during the study or had a special disease (needing to take drugs with the complication of gingival overgrowth), they were excluded.

**Figure 1:**
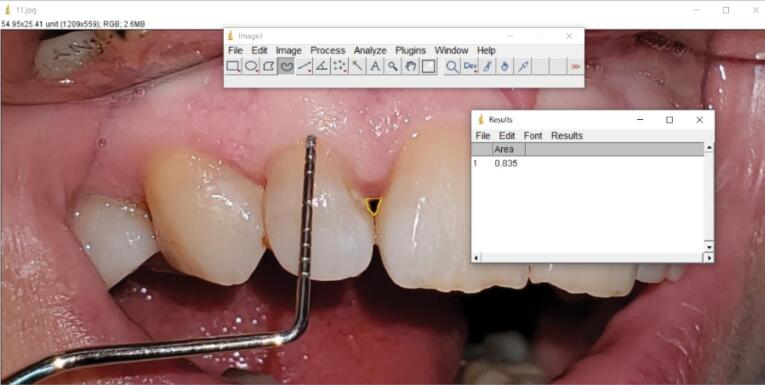
Measurement of the area of black triangles in ImageJ software and calibration by a periodontal probe (before)

**Figure 2:**
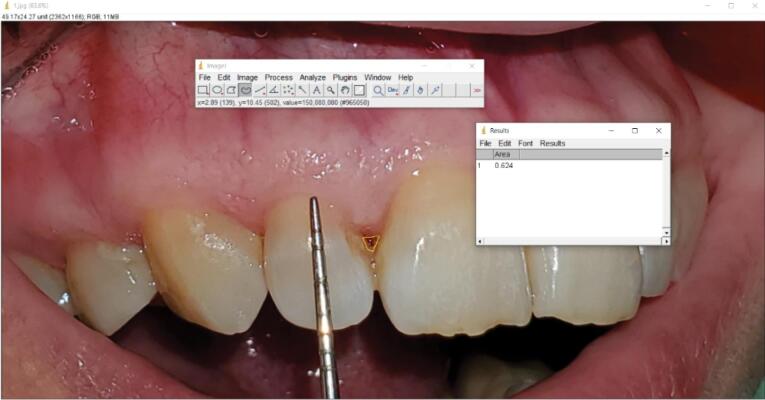
Measurement of the area of black triangles in ImageJ software and calibration by a periodontal probe (after)

 The first visit started with collecting demographic information and medical and dental histories. After signing the informed consent form, the patients received primary periodontal treatment, including scaling and subgingival debridement if needed, and the participants’ motivation for proper oral hygiene instructions was ensured. Before starting the test, the interdental papillae were evaluated according to Nordland and Tarnow’s classification.^17^ Also, clinical photographs were taken from the papillae perpendicular to the contact point. The area of the black triangles was calculated in the ImageJ software (Java 1.8.0_112 64-bit, USA) at the time intervals before the intervention and three and six months after the intervention (Figures 1 and 2). Standardization was done clinically by a Williams periodontal probe (Hu-Friedy, USA) during photography.

 The patients were randomly divided into two experimental groups: injection of Alb-PRF gel and HA gel. All the papillae belonging to each person were assigned to only one group. Finally, 25 and 21 papillae (5 volunteers in each group) were placed in the Alb-PRF and HA groups, respectively.

**Figure 3:**
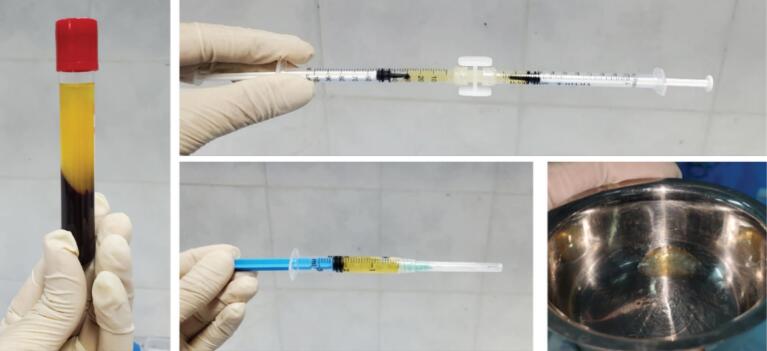
Centrifuged blood (left), mixing the denatured PPP with the liquid PRF via a sterile syringe-to-syringe connector (upper) and Alb-PRF ready to use (lower)

 In both injection stages, in the Alb-PRF gel group, 9 mL of whole blood from each patient was collected in a plastic tube and immediately centrifuged at 700 g for 8 minutes at room temperature. After centrifugation, the upper 2 mL of PPP was collected in a syringe and heated for 10 minutes at 75°C to prepare denatured albumin (albumin gel). Then, the tube was given another 10 minutes to cool to room temperature. Liquid PRF (layer on RBC), containing residual cells and growth factors, was mixed with albumin gel and Alb-PRF gel prepared for injection (Figure 3).^16^ In the other group, HA dermal filler (Revofil, South Korea®) was transferred to Loer-Lok insulin syringes (Helma Teb, Iran) with a 29-gauge needle using a female-female connector and under sterile conditions.

 The area was anesthetized (vestibular infiltration technique) with 2% lidocaine with 1:80 000 epinephrine (Darou Pakhsh, Iran). Then, < 0.2 mL of HA gel or Alb-PRF gel was injected into the papilla at a distance of 2‒3 mm apical from the tip of the papilla and at an angle of 45º to the longitudinal axis of the adjacent teeth. The patients were asked to refrain from interdental cleaning for two weeks and recalled three weeks after injection to receive a booster dose. The participants were re-examined three and six months after the first injection, and images and data were recorded. Calibration was done using a Williams probe in all photos, and the area of black triangles was calculated using ImageJ software. The Loe^18^ dental plaque index around the treated teeth was evaluated before and six months after the treatment. Also, patients’ satisfaction with esthetics was evaluated at the end of the 6-month period, using a visual analog scale (VAS) from 0 to 100 and according to the initial clinical images of the patients (0 = completely unsatisfied, 100 = very satisfied).

 The data were analyzed using SPSS 21. The normal distribution of the quantitative data of the study was measured using the Shapiro-Wilk test and the Q-Q Plot diagram. Mann-Whitney test was used to compare the changes in the area of black triangles between the two groups. Also, the changes in the area of black triangles at the desired time intervals in each group were compared using the Friedman test. A comparison of qualitative variables at the beginning of the study was made using the chi-squared test. The Wilcoxon’s signed-ranks test was used to compare the qualitative variables before and after the intervention in each group. Generalized estimating equations (GEE) were used in the software Stata 17 to compare the changes in the area of black triangles and plaque index (with adjustment of the effect of baseline value and age) and the satisfaction scores of the treatment outcomes (adjusting the effect of age) between the two groups. The significance level was defined at *P* < 0.05.

## Results

 The present study was conducted on 10 patients, including 5 patients (3 men and 2 women) with 25 incomplete papillae in the Alb-PRF gel group and 5 patients (3 men and 2 women) with 21 incomplete papillae in the HA gel group.

 The mean age of the patients was 41.24 ± 12.17 years, with a median of 38 years and a range of 24‒63 years. The mean age of patients in the Alb-PRF group was significantly higher than that of patients in the HA group (*P* < 0.001). Regarding gender ratio (*P* = 0.641) and papilla analysis class (*P* = 0.979), no statistically significant differences were observed between the two groups.

 No statistically significant differences were observed in black triangle area measurements between the two groups at the beginning of the study (*P* = 0.408) and 3 (*P* = 0.604) and 6 (*P* = 0.300) months after the intervention. In the Alb-PRF group, the area of black triangles decreased from the beginning of the study (0.061 ± 0.024) to the third month (0.036 ± 0.023) and the sixth month (0.026 ± 0.019) after the intervention. The reduction percentage of the area of black triangles in this group in the third and sixth months compared to before the intervention was 45% and 66%.

 In the HA group, the area of the black triangles decreased from the beginning of the study (0.067 ± 0.022) to the third (0.037 ± 0.017) and sixth months (0.030 ± 0.016) after the intervention, and this area reduction in the third and sixth months was 56% and 64%, respectively. These reduction changes were statistically significant in both groups (*P* < 0.001).

 The satisfaction score of the patients with the aesthetic results of the treatment in Alb-PRF and HA intervention groups was 60.00 ± 14.43 and 65.24 ± 9.42, respectively, which was higher in the HA group. However, the difference was not statistically significant (*P* = 0.219).

 The microbial plaque index accumulated on the teeth adjacent to the treated papilla six months after the intervention compared to the baseline was lower in 10 people, higher in 3, and unchanged in 12. The comparison of plaque index before and six months after the intervention in the Alb-PRF group was statistically significant (*P* = 0.046). The dental plaque index in the HA group at the end of the sixth month compared to the baseline was lower in 4 individuals, higher in 2, and unchanged in 15, which was not statistically significant (*P* = 0.414).

 Data analysis using GEE showed that in terms of the area of black triangles (*P* = 0.994) and plaque index (P = 0.566), there was no statistically significant difference after adjusting the effect of baseline values and age between the two investigated groups. Also, the satisfaction score of the treatment results was not significant after adjusting the impact of age between the two groups (*P* = 0.965).

## Discussion

 The interdental papilla is highly effective in the beauty of the anterior teeth and smile. Complaints about esthetic problems due to loss of interdental papillae are very common, especially after periodontal surgical treatments.^19-21^ Several surgical methods have been used to solve these problems and fill the black triangles. Most of these methods are very invasive and unpredictable. On the other hand, non-surgical restorative and prosthetic methods are not quite acceptable in final esthetics and cause permanent damage to the teeth.^22^ In addition, orthodontic procedures are very time-consuming and unpredictable. Nowadays, less invasive periodontal procedures have attracted attention due to the simplicity of application, greater acceptance of patients, and reduced complications. The use of non-invasive biocompatible methods, such as commercially available HA gel injection, to fill the gingival embrasure space and reduce the area of black triangles can replace conventional invasive surgical treatments; however, it is costly and may require repeated injections.^23,24^

 Castro-Calderón et al^25^ also reported in a recent systematic review that the non-surgical use of HA injection seems to positively affect the regeneration of lost interproximal papillae. However, postoperative complications may occur. Based on this, autologous replacement with a simple and low-cost processing method reduces costs and protects the patient from complications such as infection and foreign body reactions.

 The current study is the first investigation of an autologous gel prepared from patients’ blood without adding any anticoagulant, called Alb-PRF, for papilla reconstruction compared to HA. In this work, Alb-PRF was used to increase the volume of the interdental papilla in one of the groups by heating PPP at 75°C and mixing the resulting denatured albumin with liquid PRF, which was introduced with the message of “lower degradation speed and improved mechanical and rheological properties.”^16^ Before that, Jiménez Gómez et al^26^ used a new injectable gel based on plasma rich in growth factors (PRGF) technology to create long-term stability in the shape and volume of soft skin tissue. In comparing this injectable gel’s high- and low-viscosity forms, similar growth factor content provided moderate wrinkle reduction, and natural facial volume changes with 16 weeks of clinical improvement maintenance and high patient satisfaction. Considering the purpose of using autologous materials, i.e., using pure biological material without adding any other material, and the necessity of using sodium citrate in preparing PRGF, it seems more logical to use Alb-PRF.

 The high degradation rate of biological materials prepared from blood (about two weeks) prompted Zheng et al^27^ in 2022 to heat PPP at 45, 60, 75, and 90 °C to determine the most suitable temperature for PRF heat treatment. The obtained PRF gel at 75 °C for 10 minutes could produce a uniform and moldable gel with the highest resistance to degradation, excellent rheological behavior, and a high percentage of viable cells. According to the results of this study, the temperature selected in the present study is the best temperature for heat treatment to prepare Alb-PRF.

 Karimi et al^28^ used a carbodiimide crosslinker to find a way to slow down the release of growth factors and improve the properties of PRF gel. In crosslinked gels, the degradation time increased from eight days (control) to more than two weeks. According to the results of this study, the use of a crosslinker, despite improving the mechanical properties and absorption time of PRF gel, could improve the degradation time by only a little more than two weeks. In fact, heating up to 75 °C is a simple method. It is more effective in reducing the degradation rate to at least more than 25 days^25^ and does not need any additives.

 In the present study, after two injections of Alb-PRF gel at the base of the papilla with an interval of 21 days, the reduction in the area of the black triangles in the third and sixth months was 45% and 66%, respectively. The application of this gel led to the reconstruction of the interdental papilla with its unique biological properties and the ability to maintain volume for a long time with a low-cost, minimally invasive procedure. Recent studies focusing on biological materials have reported similar results.

 In a case series study by Puri et al,^15^ i-PRF was used for the first time for the non-surgical treatment of black triangles. Clinical and photographic evaluations 1, 3, and 6 months after the intervention showed that the non-surgical method of i-PRF injection successfully treated incomplete interdental papillae and resulted in patient satisfaction. In three out of six papillae, the filling rate of the papilla defect was 100%; in one area, it was 75%, and in two areas, it was 66.6%, which is consistent with the present study. Previously, Miron et al^29^ stated that i-PRF can release additional growth factors in the next 10 days by forming a dynamic hydrogel, possibly inducing tissue regeneration. Of course, due to the liquid nature of i-PRF and its high absorption rate, it still seems that using a biological gel with a slower degradation rate will yield better results. Perhaps the selection of a larger population will reduce the papilla fill results obtained. Oswal and Kour^30^ also showed similar results in a case report. By describing a new approach to strengthen the papilla and repeated injections of i-PRF in three months and follow-up up to six months, they reported stable results concerning the reconstruction of the interdental papilla, and the patients also showed good acceptance of the treatment. The results of the present study are consistent with those of a clinical trial by Ozsagir et al^14^ in 2020. In this trial, individuals with thin periodontal phenotype were randomly injected on one side with i-PRF microneedling and on the other with i-PRF in four sessions with 10-day intervals. Both interventions increased gingival thickness, and a statistically significant difference was observed in the microneedling and i-PRF groups in the sixth month. In fact, the significant clinical difference between this study and the current study is in the injection site, which, in Ozsagir and colleagues’ study, changes in volume in the horizontal dimension (gingival thickness) and the present study in the vertical dimension were investigated, which increased in both studies.

 According to the present study, two injections of HA were successful in regenerating the interdental papilla and reducing the area of black triangles. In the 3- and 6-month follow-ups, 56% and 64% reductions in interdental papilla defects were observed. In a study by Sadat Mansouri et al,^11^ six months after two injections of HA into the papillae, half of the papillae showed > 50% improvement. However, in three months, only 10% of the papillae showed improvements, and between the third and sixth months, improvements increased, which is consistent with the results of the present study. Also, the results of the present trial are similar to the results of Abdelraouf and colleagues’^23^ study, in which three injections of HA gel into the interdental papillae led to a significant reduction in the level of black triangles at both 3- and 6-month intervals, with no significant differences compared to saline injection (control group). Moreover, Firkova^31^ observed significant changes in papilla gain in the first, third, and sixth months (59%, 72%, and 77%, respectively) of two HA injections. Also, Alhabashneh et al^9^ observed a significant decrease of 39% and 29% in the height of black triangles during 3 and 6 months of follow-up, respectively, with two injections of HA in incomplete interdental papillae in the esthetic area; apparently, the increase in papilla height obtained in the third month was lost until the sixth month. However, the results in the first six months after the injection seemed promising. Perhaps the reason for this decrease in papilla height can be found in the anatomy of the embrasures, the type of injected HA, and the interdental cleaning methods. In a case report in 2020, Spano et al^32^ achieved 1.75 mm of papilla fill within six months by injecting HA into the subperiosteal space of the interdental papilla. Despite the satisfactory results obtained, due to the need for periosteal reflection to provide subperiosteal space for HA injection, it is a more invasive process than intra-soft tissue injection, and the probability of complications is higher.

 On the other hand, in the study by Sharma et al,^33^ six months after using palatal subepithelial connective tissue graft with coronally displacement flap, there was a 60% reduction in the black triangle area, which was statistically significant. Although complete reconstruction of the interdental papilla was not achieved, predictable and favorable surgical results were reported. Despite the acceptable results, it is an invasive procedure with high morbidity due to the removal of the connective tissue graft from the palate, which creates a periosteal incision in the recipient site. Moving the flap coronally while not fully reconstructing the interdental papilla questions the cost-effectiveness of such treatments. Despite the similar results in this study and the current study, using a less invasive, simple, and quick method, such as injection of an autogenous biological substance or commercial HA filler, seems more practical.

 Until now, no study has compared the effect of HA and biological materials derived from blood, such as Alb-PRF, on the augmentation of gingival soft tissue. In the present study, in each of the Alb-PRF and HA groups, the injection of the substance caused a significant decrease in the area of the black triangles after three and six months compared to the baseline, and the comparison between the two groups revealed no significant differences. The clinical study of Fedyakova et al^34^ also showed similar results regarding the use of HA gel and biological material prepared from blood (but in the skin), which indicates the suitability of these biological materials to replace commercial materials and achieve clinical results. In this study, while introducing a new injectable autogenous biological formula with favorable mechanical and bioactive properties as a safe and effective treatment for depressed skin areas, it was compared with HA as the gold standard for skin rejuvenation. This autologous gel, prepared from the patient’s peripheral blood sample in sodium citrate tubes, stimulated chemotaxis, cell migration, and collagen type I synthesis like HA. However, in terms of general biomechanical properties, it performed better than HA. It maintained the optimal viscoelastic state of the gel for strengthening soft tissue, and in addition to inducing higher cell proliferation than HA, the effect of soft tissue augmentation along with the overall improvement of skin quality in terms of hydration, elasticity, wrinkle reduction, and general satisfaction scores.

 In evaluating patients’ satisfaction with the treatment, gel injection in the interdental papilla in the Alb-PRF and HA groups was associated with 60% and 65% satisfaction, respectively, with no significant difference between the two groups. Few studies have investigated patients’ satisfaction with such treatments, and their results are consistent with the present study. In assessing patients’ satisfaction with HA injection compared to the control group (saline), in Abdelraouf and colleagues’^23^ study at the end of 6 months, HA injection was associated with greater patient satisfaction. Also, Spano et al^32^ measured the average visual analog scale (VAS) after treating interdental papilla defects with subperiosteal injection of HA and reported a 62% improvement in patient satisfaction regarding papilla filling. In the study of Puri et al,^15^ the i-PRF to reconstruct the incomplete interdental papilla successfully treated the incomplete interdental papilla and resulted in patient satisfaction.

 In assessing the amount of microbial plaque accumulated on the treated teeth in the Alb-PRF group, comparing the plaque index before and six months after the intervention was statistically significant but not statistically significant in the HA group. In comparing two groups by adjusting the effect of baseline values and age between the groups, no significant difference was observed. Considering the plaque control training at the beginning of the study and the necessity of the absence of gingivitis to be included in the intervention group, only patients who had adequate plaque control were included in the study. Since the Loe^18^ plaque index is qualitative, it is not easy to judge this variable, and further studies with quantitative and measurable indicators are necessary. None of these studies have yet evaluated any plaque index.

## Conclusion

 Based on the results of this study, the injection of HA or Alb-PRF in the defective interdental papilla may be able to restore the soft tissue to a large extent. In addition, it may lead to patient satisfaction by reducing the area of the black triangles.

## Competing Interests

 The authors declare no conflicts of interest.

## Consent for Publication

 Written informed consent was obtained from all the participants in the study.

## Data Availability Statement

 All data regarding the methodology of the manuscript have been shared.

## Ethical Approval

 This clinical trial has been approved by the ethics committee of Guilan University of Medical Sciences (IR.GUMS.REC.1400.604) and the Iranian Registry of Clinical Trials (IRCT20130813014350N4).
